# Individualized preoperative planning using three-dimensional modeling for Bismuth and Corlette type III hilar cholangiocarcinoma

**DOI:** 10.1186/s12957-016-0794-8

**Published:** 2016-02-24

**Authors:** Ning Zeng, Haisu Tao, Chihua Fang, Yingfang Fan, Nan Xiang, Jian Yang, Wen Zhu, Jun Liu, Tianpei Guan, Cheng Fang, Fei Xiang

**Affiliations:** Department of Hepatobiliary Surgery, Zhujiang Hospital of Southern Medical University, Guangzhou, 510282 China

**Keywords:** Hilar cholangiocarcinoma, Type III, Computed tomography, Three-dimensional reconstruction, Preoperative planning

## Abstract

**Background:**

A detailed evaluation of blood supply anatomy, especially the biliary anatomy at the hepatic hilus, is essential to ensure a complete and curative resection for Bismuth and Corlette type III hilar cholangiocarcinoma. The study aimed to investigate the impact of individualized preoperative planning using 3D modeling on surgical treatment for type III hilar cholangiocarcinoma.

**Methods:**

This was a retrospective study of patients with type III hilar cholangiocarcinoma (*n* = 47) who underwent surgery at the Hepatobiliary Surgery Department of Zhujiang Hospital between March 2007 and January 2015. All patients had undergone preoperative computed tomography (CT) examination, and 3D images were reconstructed. Preoperative surgery simulation was performed, and the simulation was applied in the subsequent surgery. Clinical, surgical, and pathological characteristics were compared between patients undergoing preoperative planning (*n* = 25) and those who did not (*n* = 22). Complications were examined.

**Results:**

Surgical time and blood loss were significantly smaller in patients with 3D reconstruction compared to those without. The number of bile duct orifices was correctly estimated in 14/25 (56.0 %) patients with preoperative planning. The width of the hepatic surgical margin could be measured for 18 hepatic ducts, and 17 (68.0 %) of them were pathologically diagnosed as margin-negative.

**Conclusions:**

This technique has the advantages of precise visualization of the anatomic structures and 3D assessment of biliary branches and vessels, allowing improved operative planning for the treatment of hilar cholangiocarcinoma.

## Background

Hilar cholangiocarcinoma accounts for 58–75 % of the cancers of the extrahepatic biliary duct, and its incidence shows an increasing trend, especially in Hispanic and Asian men [1, 2]. Surgical resection is the main treatment for hilar cholangiocarcinoma [1–3], but the resection rate is reported to be less than 40 % [1, 2, 4]. The reasons for non-resectability in these patients are local extensive invasion to major vessels such as the hepatic artery and the portal vein and metastases including peritoneal dissemination, liver metastases, distant lymph nodal metastases, and extra-abdominal metastases [1, 2, 4]. The anatomic features of the hepatic hilus often make it easy for hilar cholangiocarcinoma to invade major vessels [5, 6]. At the same time, hepatic duct obstruction may lead to liver function damage, while the preserved liver function and the critical residual liver volume after resection are difficult to assess accurately [1, 2, 4, 7]. Therefore, the preoperative assessment and surgical planning of hilar cholangiocarcinoma become extremely important [8].

In order to select the appropriate approach for patients with hilar cholangiocarcinoma, it is essential to determine the anatomic relationship between the tumor and surrounding vessels for each patient because the surgical procedure is determined by their relative positions, which vary from a patient to another [3, 9–11]. A recently developed 3-dimensional (3D) imaging technique allows the detection of the extent of tumor invasion and the relationship of the tumor to the vessels and bile duct system. Only a few studies have examined preoperative imaging and its relation to surgical finding [12–14]. It is well known that among the surgical protocols for hilar cholangiocarcinoma, the classical treatment for Bismuth-Corlette type III hilar cholangiocarcinoma [8, 15] is hemihepatectomy or extended hemihepatectomy plus hepatic caudate lobectomy [16–18]. The longitudinal extent of resection can be planned based on two anatomic landmarks, the posterior portal sagittal part (U point) and the right posterior portal branch (P point) [10, 19]. However, clinical experience suggests that the surgical margins are difficult to identify by simply applying the U and P points in case of portal vein variations.

In this retrospective study, an individualized preoperative surgical planning process combining the 3D reconstruction technique and classification for portal vein variations was developed and used for patients with Bismuth-Corlette type III hilar cholangiocarcinoma.

## Methods

### Patients

This was a retrospective study of 47 patients with type III hilar cholangiocarcinoma who were selected and who had undergone surgical treatment at the Hepatobiliary Surgery Department of Zhujiang Hospital between March 2007 and January 2015. Inclusion criteria were (1) postoperative histopathological diagnosis of type III hilar cholangiocarcinoma; (2) intraoperative diagnosis confirmed the preoperative imaging results of type III hilar cholangiocarcinoma; (3) absence of intrahepatic and extrahepatic extensive metastases; (4) absence of severe invasion of major vessels (main hepatic artery and main portal vein); (5) Child-Pugh grades A or B; and (6) the liver remnant presented more than 50 % of the functional liver volume according to computed tomography (CT) volumetric assessment.

Before surgery, all patients were informed of the treatment details including procedure, risks, and complications. Therefore, they were grouped according to whether they accepted preoperative planning using the 3D reconstruction technique (*n* = 25) or not (*n* = 22). All surgeries and postoperative management were overseen by the same surgical team.

The Ethics Committee of Zhujiang Hospital of Southern Medical University approved this retrospective study (2007-GDYK-006). All patients signed a risk consent form preoperatively, including acceptance to receive preoperative planning with 3D reconstruction technique. Because of the retrospective nature of the study, the need for individual consent was waived by the committee.

### Preoperative CT examination

All patients underwent enhanced CT (Philips Brilliance 256-MDCT scanner; Philips, Best, The Netherlands) to collect 2D images, which were processed using the MxliteView DICOM Viewer. Enhanced scanning was conducted in the liver, gallbladder, pancreas, spleen, and abdominal vessels in the plain scan phase, arterial phase, portal venous phase, and venous phase using parameters previously described [20].

For image segmentation and 3D reconstruction, DICOM data were imported into a self-developed abdominal medical imaging software (the MI-3DVS, developed by the authors [software copyright no.: 2008SR18798]), in which data of the liver, vessels, and intrahepatic bile duct are extracted using a 3D dynamic adaptive region growing method performed using sequential automatic segmentation. The specific procedures are the following: (1) a seed point is selected from the region of interest, and the gray average of a 3 × 3 area around the seed point is calculated as the initial value; (2) the target images are segmented by adjusting the upper and lower thresholds until the liver and its internal bile duct, hepatic artery, hepatic vein, portal venous system, and peripheral spleen, pancreas, gallbladder, stomach, duodenum, and other major organs are segmented from the CT images accurately and independently; (3) and 3D reconstruction is performed using matching cubes algorithm in surface rendering, which is automatically registered to obtain 3D models of the corresponding structures [20, 21].

### Image processing

The reconstructed 3D model was imported into the FreeForm Modeling System (SensAbleTechnologies, Inc., Woburn, MA, USA) for processing in order to obtain smooth, lifelike, and stereoscopic 3D models. The liver, hepatic vein, hepatic artery, portal vein, bile duct, stones, abdominal vessels, and peripheral organs were colored with different colors. Different degrees of hyalinization were performed to hide the other organs in order to observe the 3D anatomic relationships for different combinations of liver and hepatic artery, liver and portal vein, liver and hepatic vein, liver and tumors, etc. Then the 3D model was rotated to observe the spatial anatomic relationships between the lesions and the adjacent organs from different perspective. In this study, we focused on the 3D spatial relationships of the tumors with the hepatic bile duct, hepatic artery, portal venous system, and hepatic venous system.

### Classification of portal venous anatomy for type III hilar cholangiocarcinoma

**Fig. 1:**
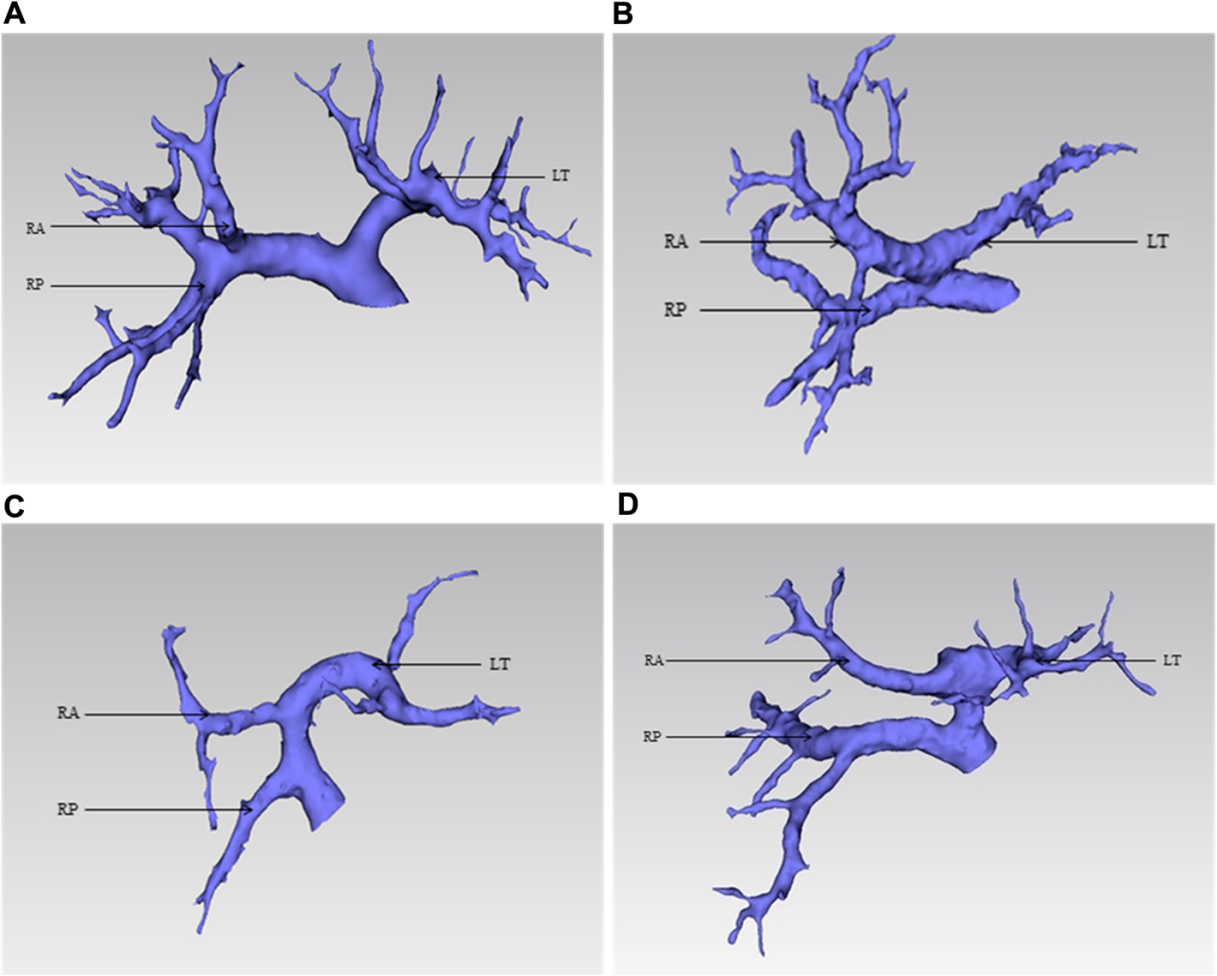
Classification system for type III hilar cholangiocarcinoma based on the 3D modeling of the portal vein. **a** Type I. **b** Type II. **c** Type III. **d** Type IV. *RA* right anterior portal vein, *RP* right posterior portal vein, *LT* left portal vein

Type I, also known as Cheng’s type I or the normal type, is presented in Fig. 1a. For right hemihepatectomy, the limit point of the left bile duct dissection was located at B2 and B3 at the left margin of the posterior portal sagittal part (U point), while the limit of bile duct dissection was located around the bifurcation of the right anterior portal branch and right posterior portal branch (P point) for the left hemihepatectomy.

Type II (Fig. 1b), also known as Cheng’s type II, features the right anterior portal vein (RAPV), right posterior portal vein (RPPV), and left portal vein (LPV) that trifurcates from the main portal vein (MPV). Since the left main portal vein is still there, the U point can be identified and the same method as that for type I can be applied for right hemihepatectomy for Bismuth-Corlette type IIIa hilar cholangiocarcinoma. However, in performing left hemihepatectomy for Bismuth-Corlette type IIIb hilar cholangiocarcinoma, the P point was moved forward to the porta hepatis, where it was close to the hilar branch due to a short right branch. Thus, the right anterior and posterior portal veins were dissected, and the hilar plate was descended in order to block and protect the right hepatic duct. The limit point of the right hepatic duct was located at the bifurcation of the right anterior and posterior portal veins.

The features of type III (Fig. 1c) include the following: (1) the right posterior portal vein (RPPV) comes directly from the main portal vein (MPV) and the left portal vein (LPV) and right anterior portal vein (RAPV) are presented as a common trunk. For right hemihepatectomy, the LPV and RAPV were dissected, and the RAPV could only be cut off after the LPV was protected. The U point was still the limit point for the dissection of the left hepatic duct. For left hemihepatectomy, the LPV and RAPV were dissected, and the LPV could only be cut off after the RAPV was protected. At this time, it was needed to continue to dissect the right liver and the hepatic plate was descended for blocking the right hepatic duct and RPPV. The limit point of the right bile duct dissection was the bifurcation of the right anterior and posterior branches of the right hepatic duct.

Type IV (Fig. 1d), also known as Cheng’s type III, features the RPPV directly coming from the MPV and the RAPV coming from the LPV at or near the umbilical point. For right hemihepatectomy, the LPV and RAPV were dissected, and the RAPV was cut off only after blocking the LPV, while the U point was still the limit point of the left bile duct dissection. For left hemihepatectomy, the LPV and RAPV were dissected. Under this circumstance, it was generally needed to split part of the liver in order to dissect the RAPV, during which the splitting of the liver was conducted as close as possible to the left side in order to protect the RAPV. If it was required to continue to dissect the right liver, the hepatic plate was descended for blocking the right hepatic duct and RPPV, and the limit point of the right bile duct dissection was the bifurcation of the right anterior and posterior branches of the hepatic duct.

### Example of individual 3D anatomic planning

**Fig. 2:**
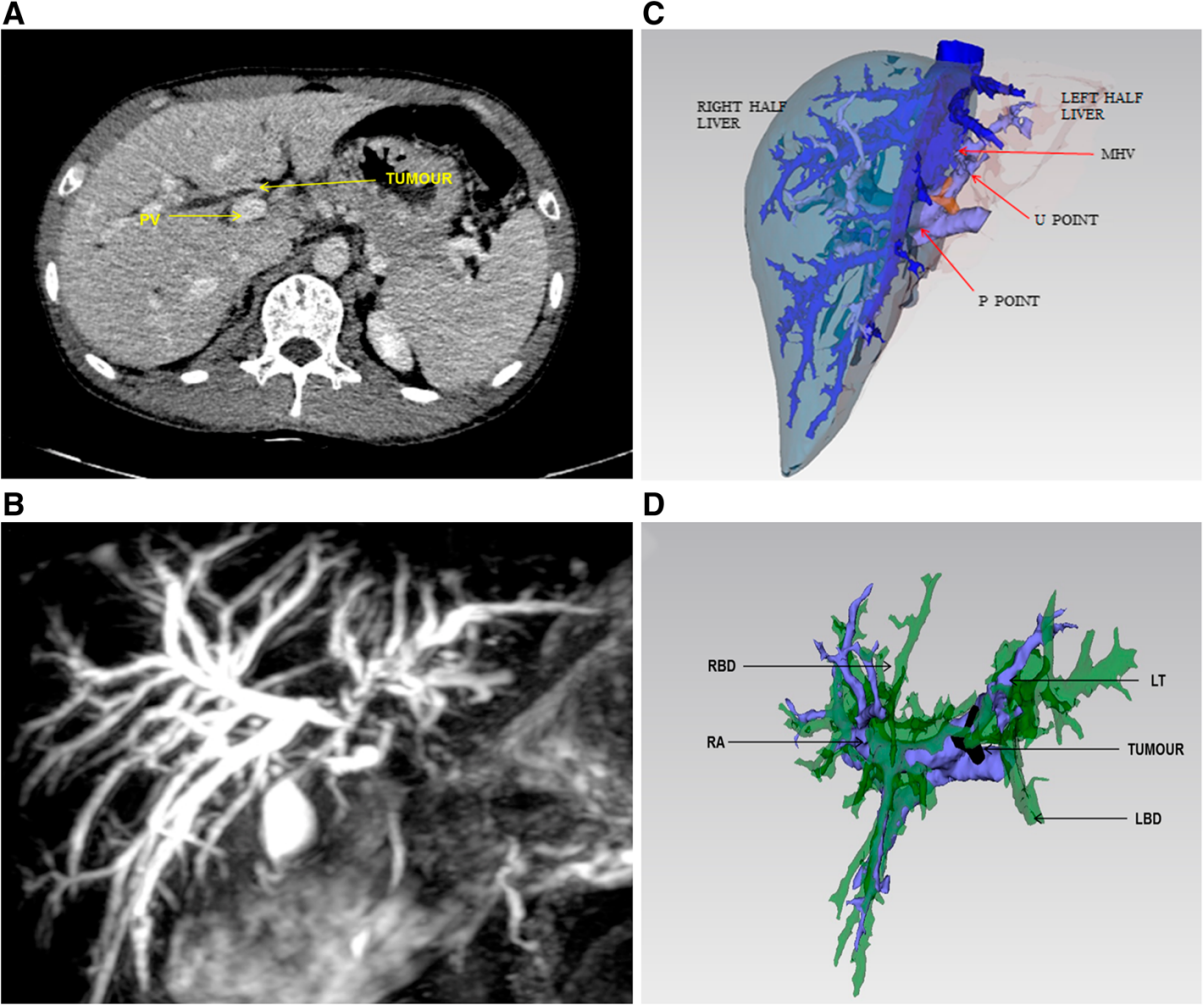
Case study of a single patient with preoperative 3D reconstruction. The patient was a 47-year-old male patient who had been suffering from yellowish urine and yellow sclera for 1 month. Preoperative TBil was 174 μmol/L and dbil was 147 μmol/L. **a** CT revealed that the left hepatic duct was mainly involved by the tumor, and the patient was accompanied by obstructive intrahepatic duct dilatation. **b** 3D reconstruction of the portal vein. **c** 3D visualization of the liver segments and the relationship between the tumor and intrahepatic ducts. **d** Relationship between the tumor and portal vein after the liver was concealed. *RA* right anterior portal vein, *LT* left portal vein, *RBD* right bile duct, *LBD* left bile duct

The patient was a 47-year-old male who had been suffered from yellowish urine and yellow sclera for 1 month. Preoperative total bilirubin (TBil) was 174 μmol/L, and direct bilirubin (dbil) was 147 μmol/L. CT and magnetic resonance imaging (MRI) revealed that the left hepatic duct was mainly involved by the tumor and accompanied by obstructive intrahepatic duct dilatation (Fig. 2a, b). Preoperative 3D reconstruction revealed that the mass invasion mainly occurred in the left hepatic duct, which was confirmed as Bismuth-Corlette type IIIb by 3D reconstruction. The patients’ portal vein appeared to be Cheng’s type II variation, where the right anterior portal vein, right posterior portal vein, and left portal branch are directly to the main portal vein (Fig. 2c, d).

### Surgery planning

**Fig. 3:**
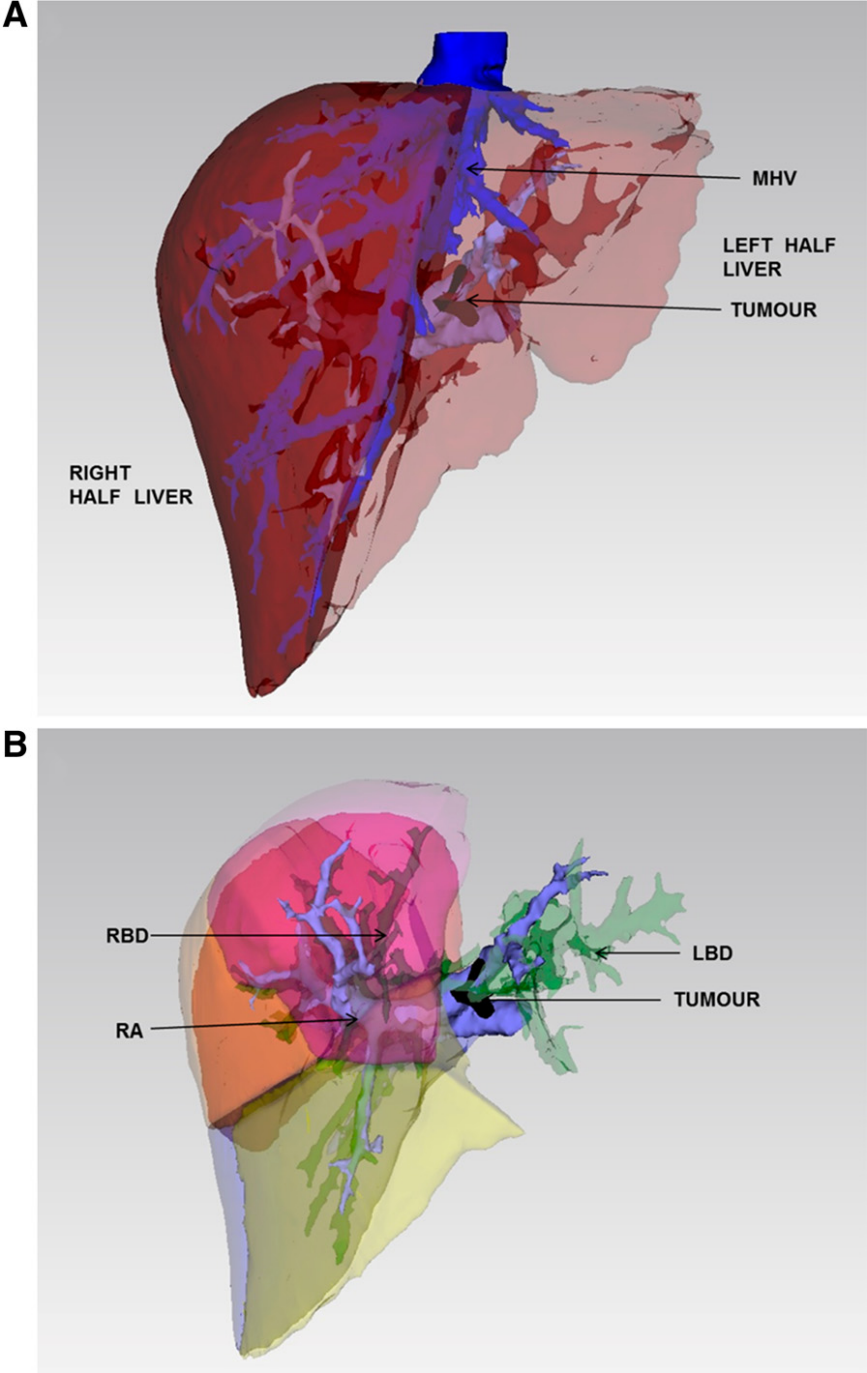
Left hemihepatectomy and hepatic caudate lobectomy were intended to be performed after preoperative operation simulation and calculation of the hemihepatectomic volume. **a** Preoperative operation simulation to calculate the volume for hemihepatectomy. **b** Operational section after left hemihepatectomy in preoperative simulation. *RA* right anterior portal vein, *LT* left portal vein, *RBD* right bile duct, *LBD* left bile duct

Surgery planning was based on the 3D reconstruction technique. The reconstructed models were exported as Standard Template Library files and imported into the FreeForm Modeling System (SensAbleTechnologies, Inc., Woburn, MA, USA), which was used to analyze the spatial distribution of the anatomic structures, hepatic artery blood supply, type of hepatocellular carcinoma, and the variations of the hepatic artery [20, 21]. The left hemihepatectomy and hepatic caudate lobectomy were performed after preoperative operation simulation and calculation of the hemihepatectomic volume (Fig. 3).

### Measurement indexes

Patient demographics and clinical characteristics were collected including age, gender, cirrhosis, bilirubin, transaminase, blood platelet, albumin, clotting time, CA19-9, CEA, Child-Pugh, number and location of tumors, PTCD, and ERCP. These surgical variables included operative time, intraoperative blood loss, number of tumors, encapsulation, satellite lesions, vascular invasion, perineural infiltration, and pathological results. Postoperative clinical outcomes included wound infection, bile leakage, abdominal infection, pleural effusion, pulmonary infection, hemorrhage, ascites, perioperative mortality, Tbil, albumin (ALB), alanine aminotransferase (ALT), hemoglobin, and CA19-9.

### Statistical analysis

Statistical analysis was performed using SPSS 20.0 (IBM, Armonk, NY, USA). Continuous data are presented as mean ± standard deviation and were analyzed using the Student *t* test. Categorical variables are presented as frequencies and were analyzed using the chi-square test. The diagnostic performance for hepatic artery (portal vein) variance between CT and 3D reconstruction was analyzed using the chi-square test. Two-sided *P* values <0.05 were considered significant.

## Results

### Characteristics of the patients

Demographics, clinical, and preoperative imaging characteristics are presented in Table 1. The two groups were similar for age and sex distributions, liver function, CA19-9, CEA, number of tumors, or proportion of patients with liver cirrhosis.

**Table 1:** Characteristics of the patients

Variables	3D reconstruction (*n* = 25)	No reconstruction (*n* = 22)	*P*
Age (years)	60.4 ± 10.4	59.0 ± 9.9	0.906
Sex, male/female	14/11	14/8	0.595
Cirrhosis, *n* (%)	17 (68.0)	15 (68.1)	0.989
TBIL (mmol/L)	37.8 ± 40.9	30.3 ± 46.5	0.788
ALT (U/L)	45.9 ± 21.9	50.9 ± 25.2	0.783
Platelets (10*9/L)	163.0 ± 69.2	161.2 ± 72.6	0.836
ALB (g/L)	34.5 ± 5.5	34.1 ± 6.1	0.445
Prothrombin time (s)	15.7 ± 2.9	15.6 ± 2.5	0.343
CA19-9, *n* (%)	16 (64.0)	16 (72.7)	0.522
CEA, *n* (%)	12 (48.0)	13 (59.1)	0.447
Child-Pugh, *n* (%)			0.510
A	9 (36.0)	10 (45.5)	
B	16 (64.0)	12 (54.5)	
Number of tumor sites, *n* (%)^a^			0.820
Single	4 (16.0)	3 (13.6)	
Multiple	14 (84.0)	15 (86.4)	
PTCD	3 (12.0)	2 (9.1)	1.000
ERCP	1 (4.0)	1 (4.5)	1.000

*TBIL* total bilirubin, *ALT* alanine aminotransferase, *AST* aspartate transaminase, *ALB* albumin, *AFP* alpha fetoprotein, *PTCD* percutaneous transhepatic cholangiodrainage, *ERCP* endoscopic retrograde cholangio-pancreatography

^a^Based on preoperative CT and/or MRI

Preoperative evaluations were performed in all patients using ultrasonography, CT, and MRI. 3D reconstruction from CT data was done for patients who accepted it.

### Intraoperative data

Table 2 presents the surgical approaches and the types of hilar cholangiocarcinoma. These variables were similar between the two groups. There were no differences in terms of number of tumors, encapsulation, satellite nodules, vascular invasion, perineural infiltration, and pathological results. Preoperative planning reduced the operative time and intraoperative bleeding (Table 3). The number of bile duct orifices was correctly estimated in 14/25 (56.0 %) patients with preoperative planning. The width of the hepatic surgical margin could be measured for 18 hepatic ducts, and 17 (68.0 %) of them were pathologically diagnosed as margin-negative.

**Table 2:** Surgical approaches

Portal vein type^a^	3D reconstruction (*n* = 25)	No reconstruction (*n* = 22)	*P*
I	17	16	0.724
II	4	3	1.000
III	2	2	1.000
IV	2	1	1.000

^a^The type of the hilar cholangiocarcinoma based on 3D model

**Table 3:** Surgical indexes and characteristics of the disease

Variables	3D reconstruction (*n* = 25)	No reconstruction (*n* = 22)	*P*
Operative time (min)	408.6 ± 65.9	447.3 ± 99.2	0.044
Intraoperative blood loss (mL)	864.8 ± 321.7	985.2 ± 549.9	0.047
Number of tumors^a^			1.000
Single, *n* (%)	21 (84.0)	18 (81.8)	
Multiple, *n* (%)	4 (16.0)	4 (18.2)	
Encapsulation, *n* (%)^a^	3 (12.0)	4 (18.2)	0.690
Satellite nodules, *n* (%)^a^	7 (28.0)	6 (19.7)	0.956
Vascular invasion, *n* (%)^a^	6 (24.0)	3 (13.6)	0.470
Perineural infiltration, *n* (%)^a^	9 (36.0)	7 (31.8)	0.763
Pathological results, n (%)			0.715
Adenocarcinoma	21 (84.0)	17 (77.3)	
Other	4 (16.0)	5 (22.7)	

^a^Based on intraoperative findings

**Fig. 4:**
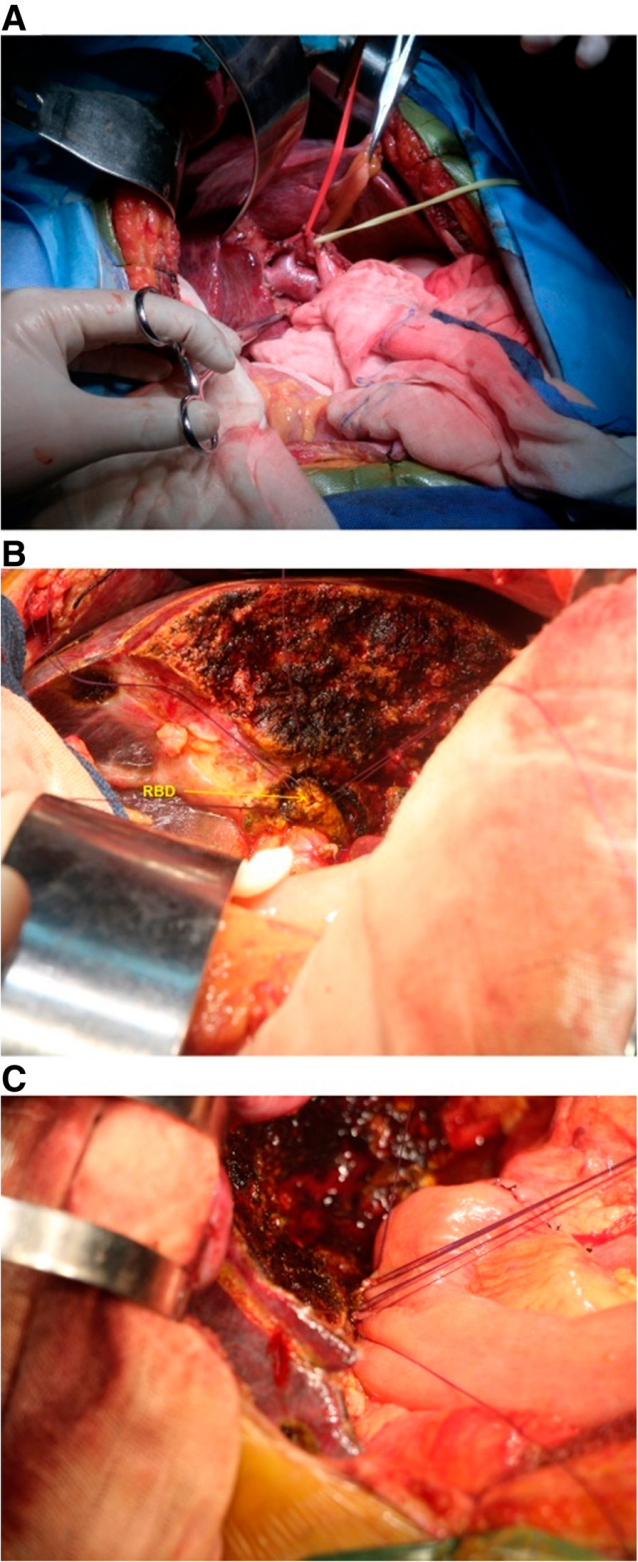
Operational procedure: **a** Preoperative anatomy of porta hepatis. **b** Hepatic section and right hepatic duct after left hemihepatectomy. **c** Postoperative superior cholangiojejumostomy

The porta hepatis was anastomized through a chevron incision at the upper abdomen, and the arteriae hepatica communis and proper hepatic artery were dissected to expose the portal vein, and then the portal vein branches and trunk were dissected and blocked, respectively. Combined with preoperative 3D reconstruction, three branches were found in the portal vein bifurcation, where one approached the right side and one approached the left side, both of which were not cut off. Dissection was continued towards the left side, and another bifurcation was found in the left portal branch, one entering the right anterior lobe and another one entering the fissure sagittalis. The left portal branch was clamped at the distal end of the right anterior branch emitting from the trunk of the left portal branch; the left liver was then significantly darkened while the right liver was of a normal color. The left portal vein was ligated and cut off, and then the left hepatic artery and left hepatic vein were cut off, achieving left hemihepatectomy combined with hepatic caudate lobectomy, clearance of hepatoduodenal ligament skeletization, right cholangioplasty, and jejunal Roux-Y anastomosis (Fig. 4a–c). The significance of the case study of a single patient is to show that 3D reconstruction can clearly show the variation of portal vein and make clear operation planning resulting in improving the safety of the operation to avoid the occurrence of complications.

### Postoperative data

There were no differences between the two groups in terms of postoperative complications such as wound infection, bile leakage, intra-abdominal abscess, pleural effusion, pulmonary infection, hemorrhage, seroperitoneum, or perioperative mortality (Table 3). On day 3, Tbil levels were decreased significantly in patients with 3D reconstruction compared to those without (*P* < 0.05).

## Discussion

Complete surgical resection is the only therapeutic strategy offering the chance of a cure to patients with hilar cholangiocarcinoma [3]. A wide resection and, sometimes, extended hemihepatectomy combined with hepatic caudate lobectomy are generally required in patients with Bismuth-Corlette type III hilar cholangiocarcinoma. A careful preoperative planning is essential to achieve good outcomes. This study examined the use of 3D modeling of the liver blood flow to plan the surgery adequately. Results showed that the number of bile duct orifices was estimated correctly in 73.7 % of patients with preoperative planning. The width of the hepatic surgical margin could be measured for 18 hepatic ducts, and 17 (94.4 %) of them were pathologically diagnosed as margin-negative. This technique has the advantages of precise visualization of the anatomic structures and multidimensional assessment of biliary branches and vessels, allowing improved operative planning for the treatment of hilar cholangiocarcinoma.

Factors for radical resection of hilar cholangiocarcinoma mainly include the following: (1) the relationship between the tumors and the limit point of bile duct dissection; (2) the blood supply of liver remnant (artery, portal vein); and (3) the volume and function of liver remnant [22]. The limit point of bile duct dissection refers to the limit part from which the bile duct can be dissected from the parallel portal vein and hepatic artery during hepatectomy, where the bile duct at the upstream of the limit point is unlikely to be dissected and cut off. The limit point of bile duct dissection is often determined according to the hepatectomy approach. For right hepatectomy, the limit point of left bile duct dissection is located at B2 and B3 at the left edge of the posterior portal sagittal part (the U point). For left hepatectomy, the limit point of bile duct dissection is located near the bifurcation of the right anterior and posterior portal branches (the P point). However, because of the variations of the anatomy of the portal vein, the preoperative assessment of these variations in relation to the tumor is essential especially for Bismuth-Corlette type III hilar cholangiocarcinoma. In addition, many patients with Bismuth-Corlette type III hilar cholangiocarcinoma also suffer from cholestatic cirrhosis and they cannot tolerate a partial hepatectomy, underlining the need for individualized surgical planning.

In this study, the Bismuth-Corlette type III hilar cholangiocarcinomas were divided into four types according to the anatomy of the portal vein and corresponding surgical protocols were developed. The 2D CT images were reconstructed into 3D images, which have some advantages compared with traditional imaging including clearly revealing the 3D anatomy of the intrahepatic and extrahepatic vessels and dilated bile duct, and a true reflection of the degree of infiltration of the tumors. The 3D images can better assess the areas supplied/drained by the different artery/vein branches, as well as the resection range or the necessity of vascular reconstruction [23].

This study showed that the use of the preoperative 3D reconstruction led to shorter surgical time and smaller intraoperative blood losses. These results are probably due to an easier localization of the tumors and a better knowledge of their interactions with the surrounding tissues. In particular, it is very convenient to know in advance the limit point of bile duct dissection and quickly identify the range of hepatectomy during the surgery according to the preoperative calculation of liver volume in cases of portal vein variations.

On day 3, Tbil levels were decreased significantly in patients with 3D reconstruction compared with those without, which might be caused by the shorter operation time and milder liver damage. Clean and tumor-free surgical margin is the target of radical hepatectomy and also is the basic condition for the prevention of recurrence. For patients with a poor liver function, it is very important to guarantee tumor-free surgical margins and also to retain normal liver tissue as much as possible.

This study suffers from some limitations. Its retrospective nature prevents exploring some data that were not routinely collected in the medical charts. In addition, the use of inclusion criteria could introduce a selection bias. Long-term follow-up was not performed to determine the oncological safety. Nevertheless, this approach could be used together with 3D printing technologies to improve the visualization of the anatomical structures. Additional studies are still necessary before implementing this technique.

## Conclusions

The use of 3D reconstruction modeling for the preoperative planning of the surgery for Bismuth-Corlette type III hilar cholangiocarcinoma has the advantages of precise visualization of the anatomic structures and 3D assessment of biliary branches and vessels, allowing improved operative planning for the treatment of hilar cholangiocarcinoma.

## Abbreviations

ALB: albumin
ALT: alanine aminotransferase
CT: computed tomography
LPV: left portal vein
MPV: main portal vein
MRI: magnetic resonance imaging
RAPV: right anterior portal vein
RPPV: right posterior portal vein
